# CRY2 mediates the cognitive decline induced by sleep deprivation in 5xFAD mice

**DOI:** 10.1371/journal.pone.0306930

**Published:** 2024-07-16

**Authors:** Sumei Luo, Lizhe Guo, Na Chen, Qulian Guo, Yongqiu Xie, Yunjiao Wang, E. Wang

**Affiliations:** 1 Department of Anesthesiology, Xiangya Hospital, Central South University, Changsha, China; 2 National Clinical Research Center for Geriatric Disorders (Xiangya Hospital), Central South University, Changsha, China; University of Nebraska Medical Center College of Medicine, UNITED STATES OF AMERICA

## Abstract

**Background:**

Cryptochrome-2 (CRY2) is a core rhythm gene that plays a crucial role in DNA damage repair. The present study investigated the potential role of CRY2 in mediating sleep deprivation-induced cognitive decline in 5xFAD mice.

**Methods:**

To assess the effects of SD on different brain regions of the mouse brain, we used ^18^F FDG PET-CT. Cognitive function was evaluated using the Morris water maze test and Y-maze. Lentivirus was used for the overexpression of CRY2, and small interfering RNA (siRNA) was used for the downregulation of CRY2 to verify the effect of CRY2. We used qRT‒PCR and Western blotting to identify the downstream factors of CRY2 and evaluated the cognitive function of mice to confirm the effects of these factors.

**Results:**

The AD mice exhibited cognitive decline after 21 days of SD and had higher expression of CRY2 compared to AD mice with normal sleep. Overexpression of CRY2 led to decreased cognitive function in AD mice, and the downregulation of CRY2 attenuated the SD-induced cognitive decline in AD mice. CRY2 reduced the expression and function of CISH, which reduced the inhibition of STAT1 phosphorylation and led to synaptic dysfunction. CISH overexpression attenuated the impairing effect of sleep deprivation on cognitive function in AD mice. Furthermore, ^18^F FDG PET-CT revealed that SD significantly reduced glucose metabolism in different brain regions of AD mice.

**Conclusion:**

Our study demonstrated that sleep deprivation upregulated CRY2 in the hippocampus of AD mice, which resulted in synaptic dysfunction by decreasing CISH-mediated STAT1 phosphorylation.

## 1. Introduction

Alzheimer’s disease (AD) is a common dementia disease, accounting for 50–70% of dementia [1], mainly manifesting as slow memory deficits associated with the loss of neuronal cells and synapses [2]. Studies have indicated that up to 50% of individuals with AD experience some type of sleep disturbance [3]. Sleep disorder is associated with the presence of amyloid deposition in the preclinical stage of AD [4], contributes to cognitive decline, and might also increase the risk of Alzheimer’s disease dementia [5]. Sleep disorder also altered the expression of circadian clock genes and aggravated Alzheimer’s disease neuropathology [6], but the precise mechanisms that sleep disorder led to cognitive decline in AD patients are unknown.

Cryptochromes (CRYs) are light-independent transcriptional suppressors involved in the transcription-translation feedback loop that generates circadian rhythms in mammals [7]. Cryptochromes, clock, bmal1, and per form the core genes of the biological clock and affect metabolic, cardiovascular, psychiatric, and sleep-phase disorders [8]. CRYs affect the synthesis and degradation of many proteins and the phosphorylation and dephosphorylation of many phosphorylation sites to maintain protein homeostasis by suppressing temporal variation in proteome composition [9].

CRYs are comprised of CRY1 and CRY2, which have similar but independent functions. Both proteins are associated with familial sleep delay [10] and interact with the glucocorticoid receptor (GR) in a ligand-dependent manner [11]. However, CRY2 has a more specific role in transcriptional regulation compared to CRY1, with a greater effect on gene expression in the cerebellum. CRY2 also plays a crucial role in DNA damage checkpoint control and regulates important cell cycle progression genes [12]. Knockdown of CRY2 significantly reverses miR-27a-3p inhibitor-induced cell apoptosis [13]. Given the vital role of CRY2 in the brain, we hypothesize that CRY2 plays a critical role in sleep deprivation to exacerbate cognitive dysfunction in Alzheimer’s disease (AD).

The Janus kinase/signal transducers and activators of transcription (JAK/STAT) signaling pathways are universal and essential to cytokine receptor signaling [14]. These pathways are comprised of four tyrosine kinases (JAK1, JAK2, JAK3, and TYK2) and seven transcription factors (STAT1, STAT2, STAT3, STAT4, STAT5A, STAT5B, and STAT6). The JAK/STAT pathway also plays a significant role in the pathogenesis of AD [15]. SOCS are the inhibitory proteins of the JAK-STAT pathway and include eight members, SOCS1–SOCS7 and cytokine-inducible SH2 domain protein (CIS), which affect neuronal differentiation and neurite outgrowth [16, 17].

The present study investigated the regulatory role of CRY2 in sleep deprivation exacerbation of cognitive dysfunction and the potential effects of the SOCS/STAT pathway on the regulation of CRY2 in AD mice.

## 2. Materials and methods

### 2.1. Animals

In all animal experiments, the National Institutes of Health Guide for the Care and Use of Laboratory Animals was followed (NIH Publication No. 8023, revised 1978). Adult male C57BL/6 mice (weighing 21-23g) were purchased from Hunan SJA Laboratory. 5xFAD mice were provided by Shen Laboratory (School of Life Science, University of Science and Technology of China). We kept the mice in a colony room at controlled temperatures (19°C–22°C), humidity (40%–60%), and light/dark cycles of 12 h/12 h with ad libitum access to food and water. We used PCR to genotype the 5xFAD mice with the following primer pairs: forward primer (5’-AGG ACT GAC CAC TCG ACC AG-3’) and reverse primer (5’-CGG GGG TCT AGT TCT GCA T-3’). All mice if needed were anaesthetized by inhalation of 4% to 6% (v/v) sevoflurane (Hengrui Pharmaceuticals, China), and anesthesia was maintained with 2% (v/v) sevoflurane. At the end of the study mice were deep anesthetized with 6% (v/v) sevoflurane and sacrificed without suffering. Then the brain tissue was taken 5 minutes after perfusion from the heart with PBS.

### 2.2. Sleep deprivation

The sleep deprivation (SD) procedure was performed following the method of Xue and Rong [18]. Briefly, nine round platforms, each with a diameter of 2.5 cm and a height of 5 cm, were evenly placed in a water tank, with equal distance between platforms. Four to six mice were placed in the tank at a time and allowed to move freely on the platforms. The water level was maintained at a depth of 1 cm below the platform surface. Mice were subjected to 20 hours of sleep deprivation in the modified multiple platform water tank, followed by 4 hours of normal sleep in their regular cages daily. The sleep deprivation period started at ZT 4 (8:00 am as ZT0) and continued until ZT 24, with normal sleep allowed from ZT 0 to ZT 4. The sleep deprivation protocol lasted for 21 days, and the water in the tank was changed daily. Throughout the study, mice had free access to food and water. After the sleep deprivation ended, the mice were given PET/CT tests and behavioral tests.

### 2.3. ^18^F-FDG-PET/CT

^18^F-FDG is a radiolabeled glucose analog that is metabolized in the same manner as glucose in blood and tissues, and it is rapidly distributed throughout the body after administration. Under normal conditions, glucose is the primary energy source of the brain, and abnormal changes in ^18^F-FDG uptake in the brain are used as diagnostic indicators of AD, PD, and HD. The following specific procedure was used: mice were anesthetized via 2% isoflurane inhalation, and 200±10 μCi of ^18^F-FDG was injected into the tail vein. The imaging began 60 minutes later.

### 2.4. Behavioral assessment

#### 2.4.1. Morris water maze (MWM) test

In order to prepare the mice for behavioral testing, they were adapted to the testing room for 30 minutes after undergoing SD for 21 days. The MWM test was performed as previously described [19], with a 5-day training phase and a probe test on the 6th day. During the training phase, mice were given one minute to find the platform. If a mouse successfully found the platform within 1 minute and remained there for 3 seconds, then the experiment was terminated. If not, the mouse was guided to the platform and allowed to stay for 20 seconds‥ In the probe test, the platform was removed, and the mice were allowed to swim for one minute. Computerized tracking systems (Smart v3.0, Panlab, Spain) were used to record and analyze their movement.

#### 2.4.2. Y-maze novel arm test

As previously described, the Y-maze novel arm test was conducted [20]. There were three arms and a center in the maze. Mice were placed in one open arm facing the wall, while one arm was closed prior to the test. In the maze, mice explored the two open arms for 8 minutes.After 30 minutes, the mice were then returned to the Y-maze for five minutes with all arms open A record was kept of the time spent in the novel arm and the number of times it was entered.

### 2.5. Lentiviral system

Lentiviral vectors containing the CRY2 sequences (LV-CRY2) and the corresponding control lentivirus (LV-NC) were purchased from HonorGene (Changsha, China). BV-2 cells were transfected with lentivirus, and successful transfection was confirmed by detecting green fluorescent protein under a fluorescence microscope (Leica, Germany). The overexpression efficiencies of LV-CRY2 were determined using qRT-PCR.

For in vivo experiments, LV-CRY2 and LV-NC were transfected into the hippocampus of 7-week-old 5xFAD mice. We anesthetized mice with 1% sodium pentobarbital (10 μl/g body weight) and fixed them into a stereotaxic frame (RWD, Shenzhen, China). Each of the left and right DG regions of the hippocampus was injected with 1 μl lentivirus, the injection rate was 200 nl/min, and the injection point was anteroposterior (AP) −1.8, mediolateral (ML)±1.5, dorsoventral (DV) −1.8. The transfection efficiency was evaluated 3 days after lentivirus injection by monitoring GFP expression in mouse brains using a fluorescence microscope (Leica, Germany). Sleep deprivation began on the third day after virus injection.

### 2.6 Small interfering RNA

Small interfering RNA (siRNA) targeting mouse CRY2, along with a scrambled negative control, were purchased from RiboBio (Guangzhou, China). The siRNA was stereotaxically injected into the DG of the hippocampus in mice, as previously described for LV-CRY2 injections. The expression of CRY2-siRNA was confirmed using qRT-PCR and Western blotting. Sleep deprivation began on the third day after siRNA injection.

### 2.7 Adeno-associated viruses

CISH protein expression was upregulated via adeno-associated virus transfection (AAV-CISH). AAV-CISH and corresponding control adeno-associated viruses (AAV-NC) were purchased from BrainVTA (Wuhan, China). The AAV was stereotaxically injected into the DG of the hippocampus in 4-week-old 5xFAD mice. We anesthetized mice with 1% sodium pentobarbital (10 μl/g body weight) and positioned them onon stereotaxic frame (RWD, Shenzhen, China). The left and right DG region of the hippocampus was injected with 1 μl of AAV at a rate of 200 nl/min [anteroposterior (AP) −1.8, mediolateral (ML)±1.5, dorsoventral (DV) −1.8]. The transfection efficiency was evaluated three weeks after AAV injection by monitoring GFP expression in mouse brains with a fluorescence microscope (Leica, Germany). The efficiency of AAV-CISH was confirmed by Western blotting. Sleep deprivation began at 7 weeks old.

### 2.8. Immunofluorescence

Freshly collected mouse brains were fixed in 4% PFA overnight, dehydrated in 15% and 30% sucrose, and sliced into 7-μm sections. The sections were incubated with primary antibodies against IBA1 (1:200, Wako, Japan), CRY2 (1:200, ThermoFisher, Massachusetts), and CISH (1:200, ThermoFisher, Massachusetts). We incubated the samples with with Alexa Fluor 488-conjugated donkey anti-goat and donkey anti-rabbit secondary antibodies (1:200, Abcam) for 2 hours at room temperature in the dark. Images of the sections were taken with a microscope (Leica, Germany).

### 2.9. Western blot

We performed Western blotting as previously described [25]. The blots were incubated with the following primary antibodies: anti-β-actin antibody (1:8000, Affinity, 60,004–1-Ig), anti-CRY2 antibody (1:1000, ThermoFisher, Massachusetts), anti-CISH antibody (1:1000, ThermoFisher, Massachusetts), anti-STAT1 antibody (1:1000, Cell Signaling Technology, Boston), anti-phospho-STAT1 (Tyr701) antibody (1:1000, Cell Signaling Technology, Boston), anti-STAT3 antibody (1:1000, Cell Signaling Technology, Boston), anti-phospho-STAT3 (Tyr705) antibody (1:1000, Cell Signaling Technology, Boston), anti-STAT5 antibody (1:1000, Cell Signaling Technology, Boston), anti-phospho-STAT5 (Tyr694) antibody (1:1000, Cell Signaling Technology, Boston), anti-STAT6 antibody (1:1000, Abcam, UK), and anti-phospho-STAT6 (Y641) antibody (1:1000, Abcam, UK).

### 2.10. Quantitative PCR

We extracted total RNA from the hippocampus using Tranzol (TRANS, China) in accordance with the manufacturer’s instructions.We determined RNA concentrations with a NanoDrop spectrophotometer (Thermo), and synthesized cDNA using the SureScript First-Strand cDNA synthesis kit (Genecopoeia). We performed real-time RT-PCR using a 96-well microfluidic card, and we measured gene expression using SYBR Green reagent assays (Applied Biosystems, ThermoFisher Scientific). The primer sequences used for real-time PCR are provided in Table 1.

**Table 1:** Primers sequences for real‐time PCR.

	Forward (5′‐3′)	Reverse (5′‐3′)
**CRY2**	GCTAGAGTGACGGAGATGCC	TCAGGAGTCCTTGCTTGCTG
**SOCS1**	GGTTGTGGAGGGTGAGATGC	CCTGAGAGGTGGGATGAGGT
**SOCS2**	GTCTGCGAGAGACTTTGCCA	ACAGGGTACCCCAAAACGAG
**SOCS3**	TTTCCAACACCGAAGGGAGG	AAACCTGGGCTCCAAGATGG
**SOCS4**	AACAATCCGTGCTACTGGGG	TTCCTTCCAGCAGAGCTTCG
**SOCS5**	GTCCGCTGTTCCTCAGACAA	TTGCCTCCCTCAGTTGTCAC
**SOCS6**	GCCTGGGGTTGCTAGAGTTT	ACCATGGGGCCACTACTTTG
**SOCS7**	CGAGAAAGCGCCCTCATTTG	TCAGAGTGGAAAGCGAACCC
**CISH**	ACGTTCTCCTACCTTCGGGA	TAGGAATGTACCCTCCGGCA

### 2.11. Statistical analysis

Behavior test results are presented as means + SEM, while other data is presented as means + SD.We used GraphPad Prism 8.0 to analyze our results. For group comparisons, one-way or two-way ANOVA were used, with P < 0.05 considered significant‥

## 3. Results

### 3.1. SD decreased the glucose absorption rate in the hippocampus of AD mice

**Fig 1:**
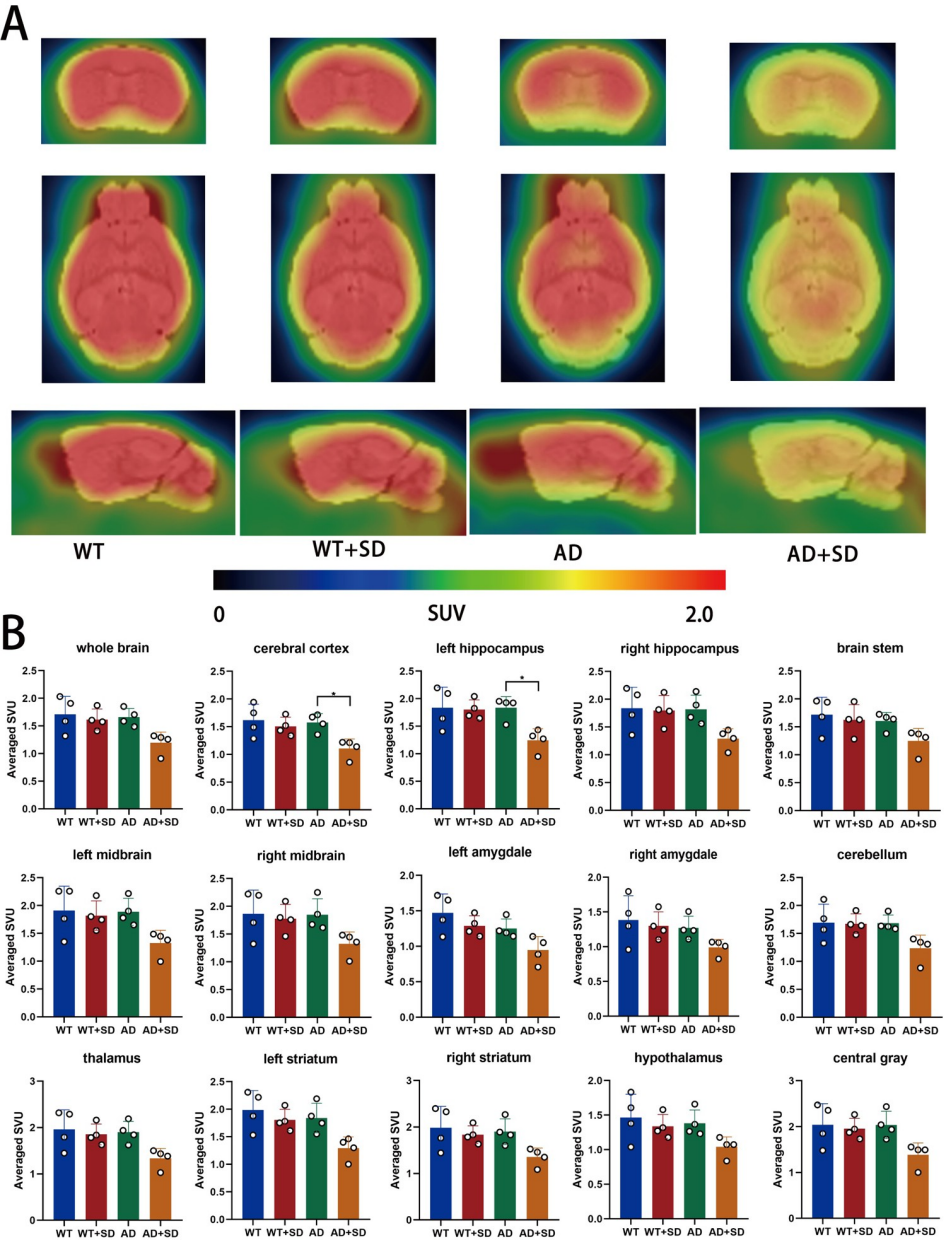
Sleep deprivation leads to a reduction in the rate of glucose absorption in the hippocampus of AD mice. Panel A displays PET/MRI template fusion images in the coronal, sagittal, and horizontal planes. The scale bar indicates that the PET image color ranges from black to red, and the SUV threshold ranges from 0 to 2.0. In Panel B, the SUV of various brain regions is presented as the means ± SD, with statistical analysis performed using ANOVA and Tukey’s test (*p < 0.05). The study included four mice in each group (n = 4).

Changes in glucose metabolism are closely associated with Alzheimer’s disease. In Alzheimer’s disease patients, glucose metabolism is significantly decreased and Aβ expression is elevated [21, 22]. Changes in rhythm genes can lead to abnormal glucose metabolism in brain tissue [23, 24]. In our study, ^18^F-FDG PET/CT were performed on mouse brains after 21 days of sleep deprivation (SD). Preliminary visual analysis indicated no significant difference between the WT group and the WT+SD group in the standard uptake value (SUV). However, the SUV was decreased in mice in the AD+SD group compared to mice in the AD group (Fig 1A). To determine the specific brain regions affected by SD, we analyzed the SUVs of different brain regions, including whole brain, the cerebral cortex, left hippocampus, right hippocampus, brainstem, left midbrain, right midbrain, left amygdala, right amygdala, cerebellum, thalamus, left striatum, right striatum, hypothalamus, and central gray (Fig 1B). Our findings showed a significant decrease in SUV in the cerebral cortex (n = 4, p = 0.030) and left hippocampus (n = 4, p = 0.029) of the AD+SD group compared to the AD group [23, 24].

### 3.2. SD leads to cognitive decline in AD mice

**Fig 2:**
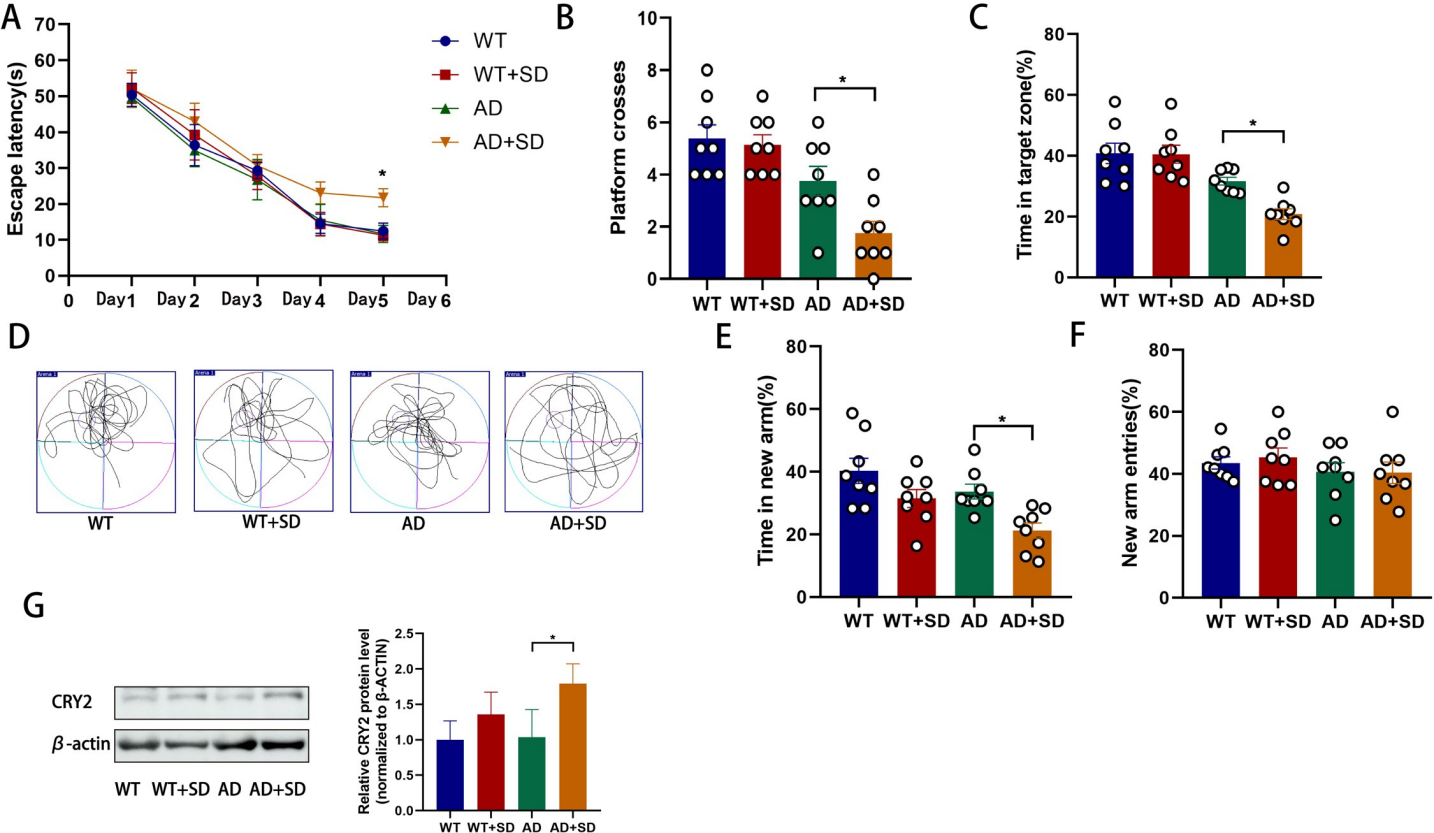
Sleep deprivation decreases learning and memory ability in AD mice and increases CRY2 expression. Data from the Morris water maze (A-D) and Y maze (E-F) were recorded and analyzed. (A) shows the latency during the acquisition phase (means ± SEM, two-way repeated measures ANOVA and Bonferroni post hoc correction, *p<0.05, AD compared to the AD+SD group). (B) shows the number of animals crossing the platform. (C) shows the percentage of time spent in the target zone. (D) shows video tracks of the probe trial. (E) shows the percentage of time spent in the new arm of the Y maze. (F) shows the percentage of new arm entries in the Y maze (means ± SEM, ANOVA, Tukey’s test, *p < 0.05). n = 8 in each group. (G) Western blotting data showing CRY expression levels in the hippocampus, with the bar graph representing CRY2 expression levels in the hippocampus (means ± SD, ANOVA, Tukey’s test, n = 6, *p < 0.05).

To investigate the effect of sleep deprivation (SD) on AD mice, we assessed cognitive function using the Morris water maze and Y-maze tests. During the training phase of the Morris water maze test, there was no significant difference in escape latency between the WT and WT+SD groups (Fig 2A). However, the AD+SD group spent longer finding the platform than the AD group on the fifth day of training (n = 8, p = 0.0494; Fig 2A). There was no significant difference in platform crosses (n = 8, p = 0.9836) or time spent in the target zone (n = 8, p = 0.9998) between the WT and WT+SD groups in the probe test. Crossing the platform was more frequent in the AD group (n = 8, p = 0.0352; Fig 2B) and spent more time in the target zone (n = 8, p = 0.0227; Fig 2C) than mice in the AD+SD group. There was no significant difference in the percentage of time spent in new arms (n = 8, p>0.9999) or new arm entries (n = 8, p>0.9999) between the WT and WT+SD groups in the Y-maze test. Compared with AD+SD mice, AD mice spent more time in the new arm (n = 8, p = 0.0423; Fig 2E), but new arm entries were not significantly different between the four groups (n = 8, p>0.9999; Fig 2F). Overall, these results suggest that SD has little effect on WT mice, but it impairs spatial memory and learning ability in AD mice, which is consistent with clinical findings that SD exacerbates cognitive decline in patients with Alzheimer’s disease.

### 3.3 CRY2 expression in the hippocampus and overexpression of CRY2 aggravates cognitive decline in AD mice

Changes in the expression of CRY2 in the hippocampus after SD were examined. Western blotting results showed an increase in CRY2 expression after SD in AD mice (n = 6, p = 0.0197; Fig 2G).

**Fig 3:**
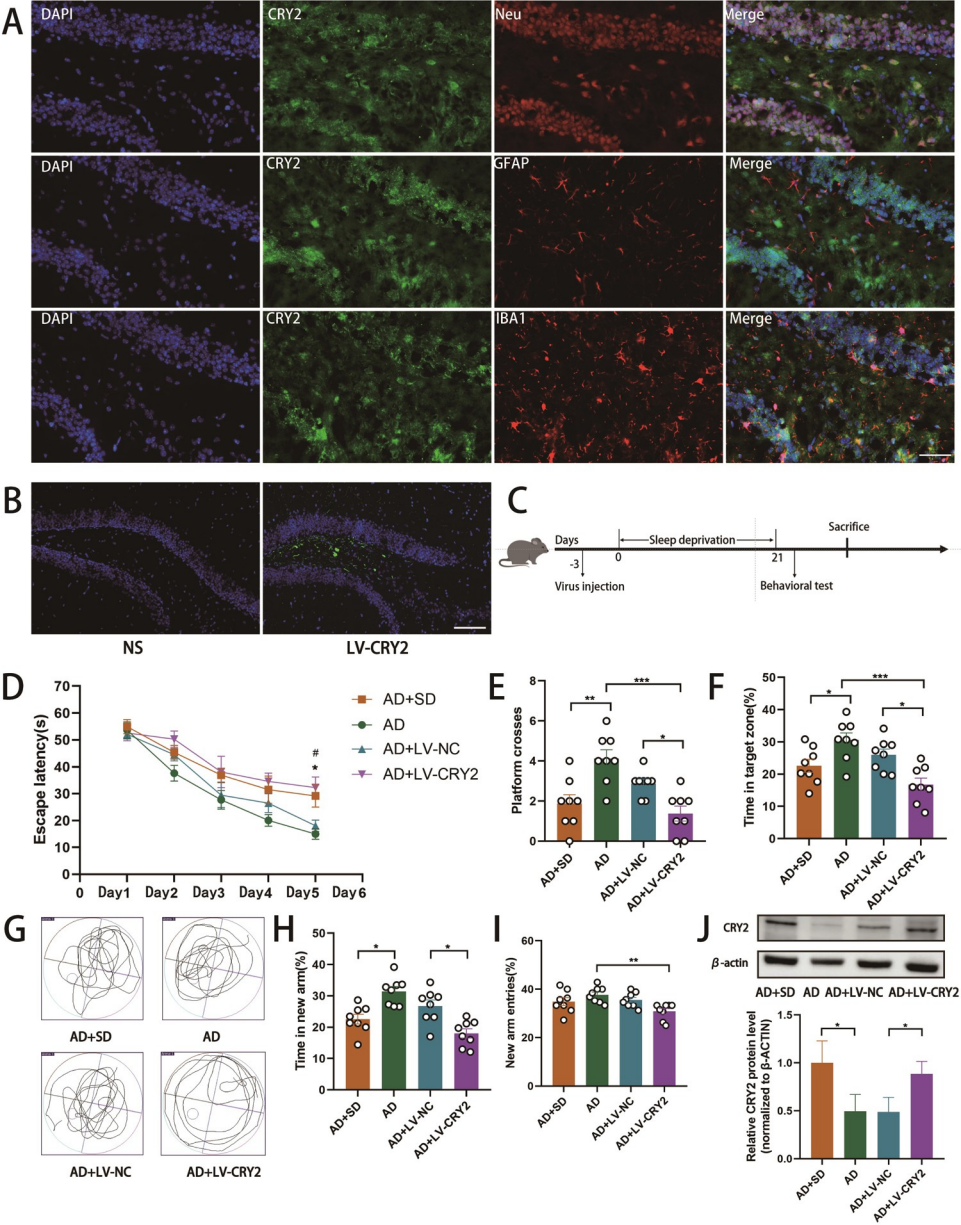
Cell-specific localization of CRY2 (A). Overexpression of CRY2 exacerbates cognitive decline in AD mice(B-J). (A) Cell-specific localization of CRY2 (green) in the DG of the hippocampus using immunofluorescence staining for neurons (red, stained with NeuN), astrocytes (red, stained with GFAP), and microglia (red, Stained with Iba1). Scale bar = 50 μm. (B) Fluorescence microscopy images of hippocampal sections injected with saline or virus. (C) Schematic representation of the experimental procedure. (D) shows latency during the acquisition phase (means ± SEM, two-way repeated measures ANOVA and Bonferroni post hoc correction, *p<0.05, AD group compared to the AD+SD group; #p<0.05, AD+LV-NC group compared to the AD+LV-CRY2 group). (E) shows the number of animals crossing the platform. (F) shows the percentage of time in the target zone. (G) shows the video tracks during the probe trial. (H) shows the percentage of time spent in the new arm of the Y maze. (I) shows the percentage of new arm entries in the Y maze (means ± SEM, ANOVA, Tukey’s test, *p<0.05). n = 8 in each group. (J) Western blotting data showing CRY expression levels in the hippocampus. The bar graph represents CRY2 expression levels in the hippocampus (means±SD, ANOVA, Tukey’s test, n = 6, *p<0.05).

To identify the cells in the DG of the HP that predominantly expressed CRY2, we performed double immunofluorescence staining. According to the results, CRY2 was primarily expressed in neurons (labeled with NeuN), and a small part was co-expressed with astrocytes (labeled with GFAP) and microglia (labeled with Iba1) (Fig 3A).

To investigate the effect of CRY2 overexpression in AD mice, we injected LV-CRY2 and observed green fluorescent protein in the DG of the HP (Fig 3B). Three days after injection, AD mice were subjected to SD (Fig 3C). Morris water maze test results showed that AD+SD mice took longer to find the platform than AD mice (n = 8, p = 0.0493). There was a longer finding time in the AD+LV-CRY2 group than mice in the AD+LV-NC group (n = 8, p = 0.0412) and the AD group (n = 8, p = 0.0131; Fig 3D). Mice in the AD group crossed the platform frequently than mice in the AD+SD group (n = 8, p = 0.0014) and the AD+LV-CRY2 group (n = 8, p = 0.0001) in the probe test. Compared to AD+LV-CRY2 mice, mice in AD+LV-NC crossed the platform more frequently (n = 8, p = 0.0443; Fig 3E). In the AD group, mice spent more time in the target zone than mice in the AD+SD group (n = 8, p = 0.0432) and the AD+LV-CRY2 group (n = 8, p = 0.0002). In the AD+LV-NC group, mice spent more time in the target zone than mice in the AD+LV-CRY2 group (n = 8, p = 0.0153; Fig 3F). As a result of the Y-maze test, mice from the AD group spent more time in the new arm in the Y-maze test than mice in the AD+SD group (n = 8, p = 0.0410) and the AD+LV-CRY2 group (n = 8, p = 0.0004). More time was spent in the new arm in AD+LV-NC mice than in AD+LV-CRY2 mice (n = 8, p = 0.0378; Fig 3H). Mice in the AD group had more new arm entries than mice in the AD+LV-CRY2 group (n = 8, p = 0.0052; Fig 3I), and the other three groups had no significant differences in new arm entries. The Morris water maze and Y-maze results suggested that overexpression of CRY2 promoted a decline in learning and spatial memory in AD mice. We measured the expression of CRY2 in the hippocampus. Western blotting results showed that CRY2 expression increased in the AD+SD group compared to the AD group (n = 6, p = 0.0115), increased in the AD+LV-CRY2 group compared to the AD group (n = 6, p = 0.0478), and increased in the AD+LV-NC group compared to the AD group (n = 6, p = 0.0423; Fig 3J).

### 3.4 Downregulation of CRY2 improves the SD-induced cognitive decline in AD mice

**Fig 4:**
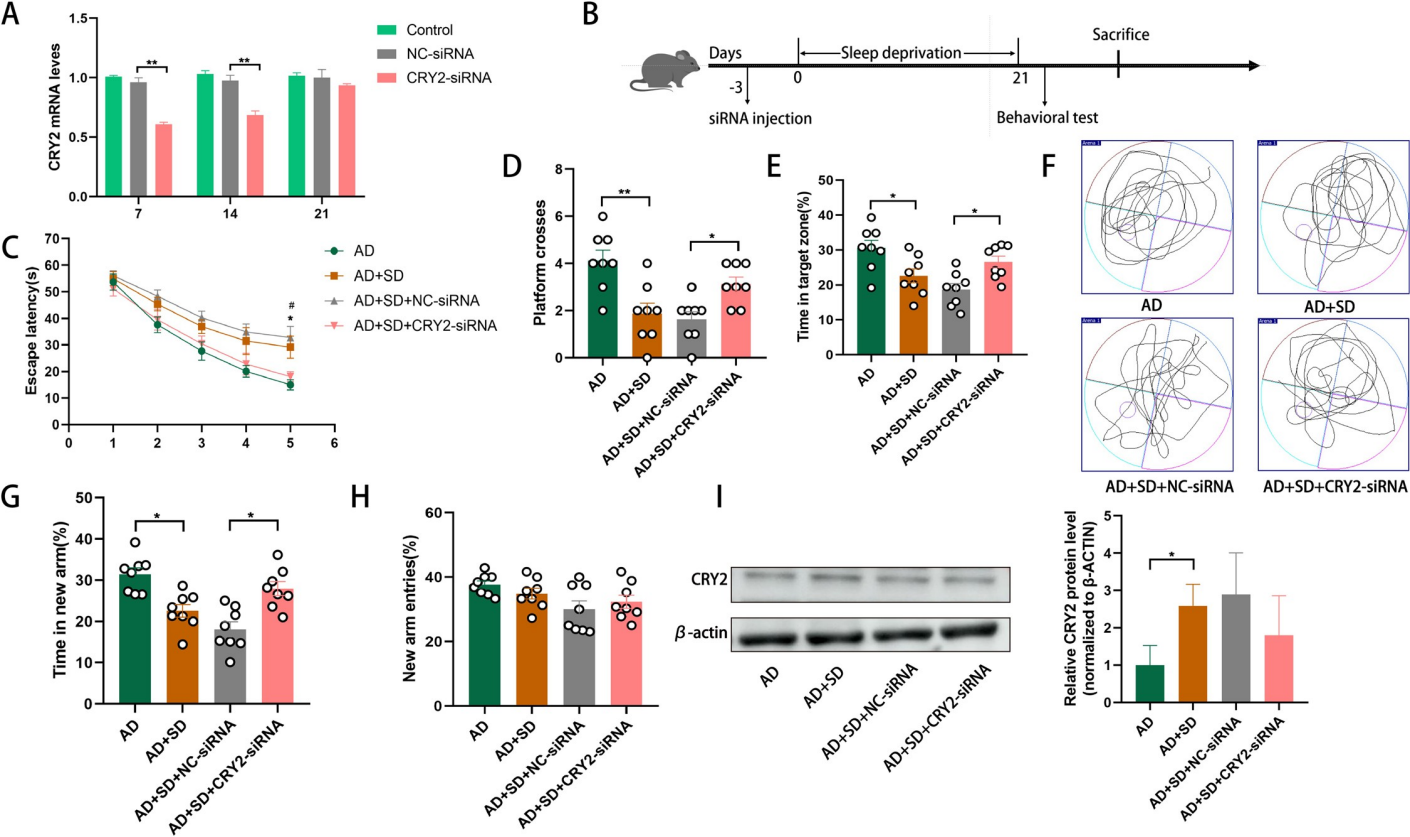
Downregulation of CRY2 improves sleep deprivation-induce cognitive decline in AD mice. (A) RT-qPCR showing the mRNA expression of CRY2 in mice after siRNA injection (means ± SD, ANOVA, Tukey’s test, n = 3, **p<0.01). (B) Schematic representation of the experimental procedure. (C) shows latency during the acquisition phase (means ± SEM, two-way repeated measures ANOVA and Bonferroni post hoc correction, *p<0.05, AD group compared to the AD+SD group; #p<0.05, AD+SD+NC-siRNA group compared to the AD+SD+CRY2-siRNA group). (D) shows the number of animals crossing the platform. (E) shows the percentage of time in the target zone. (F) shows the video tracks during the probe trial. (G) shows the percentage of time spent in the new arm of the Y maze. (H) shows the percentage of new arm entries in the Y maze (means ± SEM, ANOVA, Tukey’s test, *p<0.05). n = 8 in each group. (I) Western blotting data showing CRY expression levels in the hippocampus, and the bar graph represents CRY2 expression levels in the hippocampus (means ± SD, ANOVA, Tukey’s test, n = 6, *p<0.05).

In order to further verify the role of cry2, we investigated the effect of knockdown CRY2 on cognitive function in mice. SiRNA reduced CRY2 expression 7 days after microinjection and lasted up to 14 days. The expression of CRY2 was significantly lower in the CRY2-siRNA group than the NC-siRNA group on day 7 and day 14 (Day 7: n = 3, p = 0.0022; Day 14: n = 3, p = 0.0025). Although there was no significant difference between the CRY2-siRNA group and the NC-siRNA group on day 21 (n = 3, p = 0.3992; Fig 4A), CRY2 expression remained reduced in the CRY2-siRNA group. Three days after injection, AD mice were subjected to SD (Fig 4B). The AD+SD+NC-siRNA mice took longer to find the platform in the Morris water maze test than mice in the AD+SD+CRY2-siRNA group (n = 8, p = 0.0443; Fig 4C). A more frequent crossing of the platform was observed in the AD+SD+CRY2-siRNA group than in the AD+SD+NC-siRNA group in the probe test. (n = 8, p = 0.0443; Fig 4D). Mice in the AD+SD+CRY2-siRNA group spent more time in the target zone than mice in the AD+SD+NC-siRNA group (n = 8, p = 0.0324; Fig 4E).

AD mice treated with CRY2-siRNA spent more time in the novel arm in the Y-maze test than mice in the AD+SD+NC-siRNA group (n = 8, p = 0.0221; Fig 4G). There were no significant differences in the number of entries to the novel arm between the four groups (Fig 4H). The results of the Morris water maze and Y-maze tests suggest that the downregulation of CRY2 prevents the SD-induced decline in learning and spatial memory in AD mice.

We also examined the expression of CRY2 in the hippocampus. Western blotting analysis revealed that CRY2 levels increased in the AD+SD group compared to the AD group (n = 6, p = 0.0222; Fig 4I). Although there was no statistically significant difference between the AD+SD+NC-siRNA and AD+SD+CRY2-siRNA groups, CRY2 expression was reduced in the AD+SD+CRY2-siRNA group compared with the LV-NC group.

### 3.5. CISH is downregulated and p-STAT1 is upregulated in SD mice, and the upregulation of CRY2 reduces CISH expression and increases p-STAT1 expression

**Fig 5:**
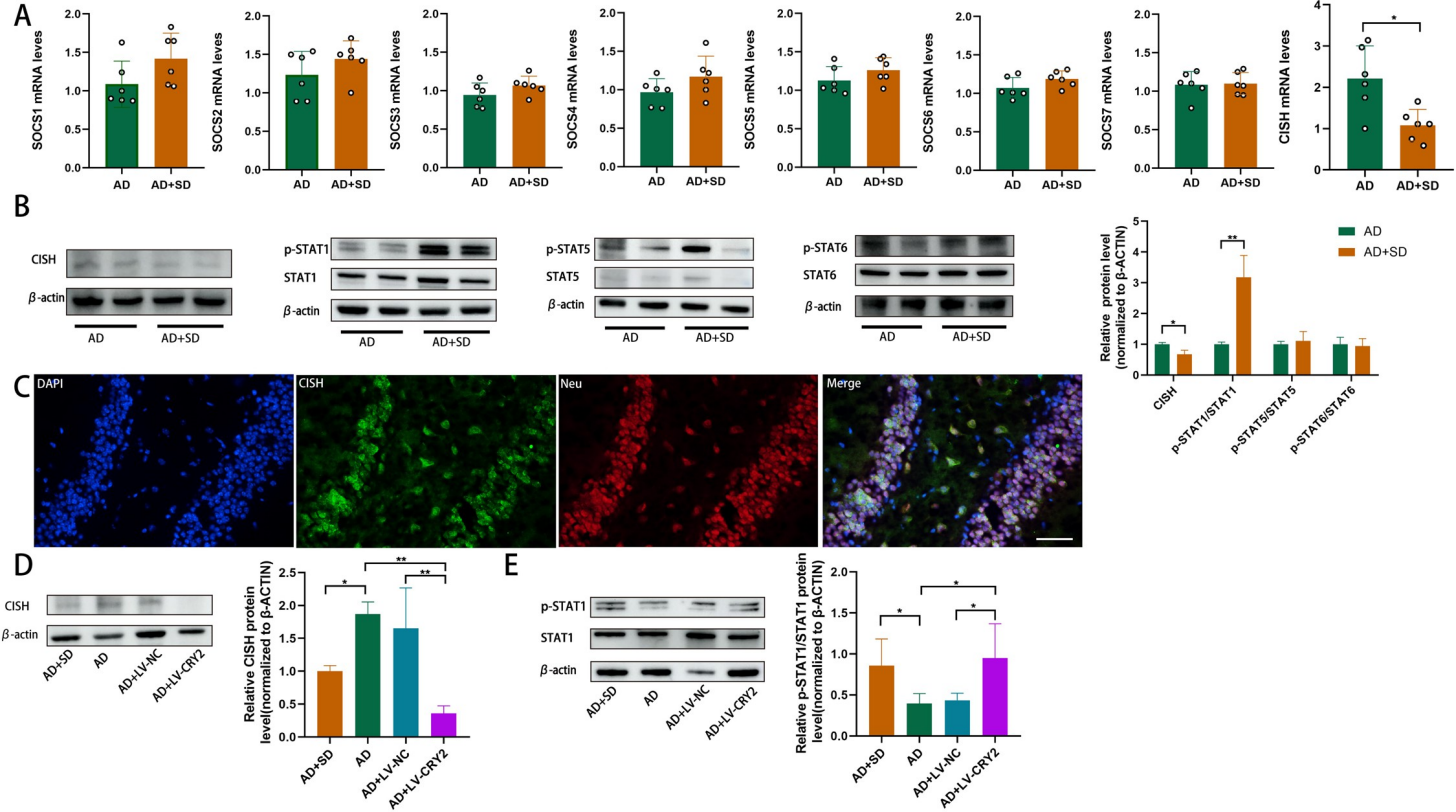
Downregulation of CISH and upregulation of p-STAT1 in the AD+SD group compared to the AD group. (A) RT-qPCR showing the mRNA expression of SOCS1-7 and CISH in the hippocampus of mice (means±SD, ANOVA, Tukey’s test, n = 6, *p<0.05). (B) Western blotting data showing p-STAT1, 5, and 6 and STAT1, 5, and 6 expression levels in the hippocampus. The bar graph represents p-STAT1 expression levels normalized to STAT1 expression levels (means±SD, ANOVA, Tukey’s test, n = 3, **p<0.01). (C) Cell-specific localization of CISH (green) in the DG of the hippocampus using immunofluorescence staining for neurons (red, stained with NeuN). Scale bar = 50 μm. (D) Western blotting data showing CISH expression levels in the hippocampus. The bar graph represents CISH expression levels normalized to β-actin expression levels (means±SD, ANOVA, Tukey’s test, n = 3, *p<0.05, **p<0.01). (E) Western blotting data showing p-STAT1/STAT1 expression levels in the hippocampus. The bar graph represents p-STAT1/STAT1 expression levels (means±SD, ANOVA, Tukey’s test, n = 3, *p<0.05).

**Fig 6:**
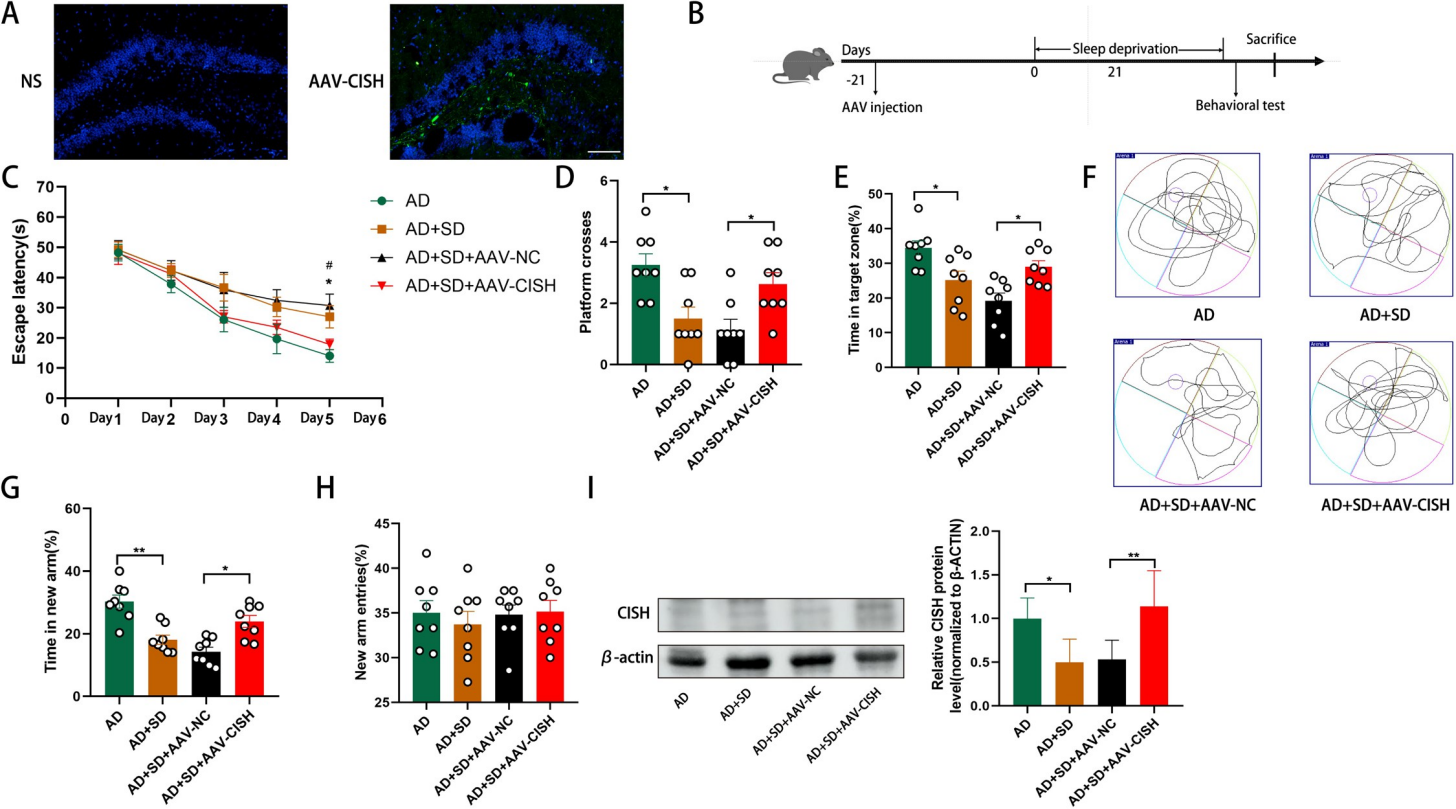
Overexpression of CISH protects against SD-induced cognitive decline in AD mice. (A) Fluorescence microscopy of hippocampal sections injected with saline or virus. (B) Schematic representation of the experimental procedure. (C) shows the latency during the acquisition phase (means ± SEM, two-way repeated measures ANOVA and Bonferroni post hoc correction, *p<0.05, AD group compared to the AD+SD group; #p<0.05, AD+SD+AAV-NC group compared to the AD+SD+AAV-CISH group). (D) shows the number of animals crossing the platform. (E) shows the percentage of time spent in the target zone. (F) shows the video tracks during the probe trial. (G) shows the percentage of time spent in the new arm of the Y maze. (H) shows the percentage of new arm entries in the Y maze (means ± SEM, ANOVA, Tukey’s test, *p<0.05, **p<0.01). n = 8 in each group. (I) Western blotting data showing CISH expression levels in the hippocampus. The bar graph represents CISH expression levels in the hippocampus (means ±SD, ANOVA, Tukey’s test, n = 6, *p<0.05).

We investigated the expression of several suppressors of cytokine signaling (SOCS) genes and CISH in the hippocampus of AD mice to elucidate the regulatory mechanism of CRY2 in SD. Compared to the AD group, the expression of CISH mRNA (n = 6, p = 0.0106; Fig 5A) and protein (n = 3, p = 0.0170; Fig 5B) was significantly decreased in the hippocampus of AD+SD mice, but there were no significant differences in SOCS1, SOCS2, SOCS3, SOCS4, SOCS5, SOCS6, or SOCS7 between the two groups. We also examined the expression of phosphorylated signal transducer and activator of transcription (p-STAT) proteins, including p-STAT1, p-STAT5, and p-STAT6. The expression of p-STAT1 was significantly increased in the hippocampus of AD+SD mice compared to the AD group (n = 3, p = 0.0060; Fig 6B). Notably, CISH was expressed in neurons (Fig 5C), which is consistent with our previous finding of CRY2 expression in neurons and suggests a potential interaction between CRY2 and CISH.

To investigate whether CRY2 upregulation alters the expression levels of CISH and p-STAT1, we compared the AD+LV-CRY2 group with the AD+LV-NC group. We observed a significant decrease in CISH expression (n = 3, p = 0.0058; Fig 5D) and a significant increase in p-STAT1 expression (n = 3, p = 0.0187; Fig 5E) in the AD+LV-CRY2 group. Our results suggest that the role of CRY2 in cognitive decline may be due to its interaction with CISH, which subsequently inhibits p-STAT1 expression.

### 3.6 Overexpression of CISH protects against SD-induced cognitive decline in AD mice

To investigate whether the upregulation of CISH protects against cognitive decline in SD-induced AD mice, we overexpressed CISH in female AD mice before SD induction (Fig 6A). Three weeks after AAV-CISH injection, the mice were subjected to SD (Fig 6B). Compared to mice in the AD+SD+AAV-CISH group, mice in the AD+SD+AAV-NC group took more time to find the platform in the Morris water maze test (n = 8, p = 0.0488; Fig 6C). Mice in the AD+SD+AAV-CISH group crossed the platform more often in the probe test than mice in the AD+SD+AAV-NC group (n = 8, p = 0.0353; Fig 6D). Mice in the AD+SD+AAV-CISH group spent more time in the target zone than mice in the AD+SD+AAV-NC group (n = 8, p = 0.0191; Fig 6E).

Compared to AD+SD+AAV-NC mice, mice in the AD+SD+AAV-CISH group spent longer in the new arm when tested on the Y-maze (n = 8, p = 0.0394; Fig 6G). A significant difference between the four groups was not found in the number of new arms entered (Fig 6H). The Morris water maze and Y-maze results suggest that CISH overexpression protects against the SD-induced decline in learning and spatial memory in AD mice.

We measured CISH expression in the hippocampus. Western blotting results showed that CISH expression increased significantly in the AAV+CISH group compared to the AAV+NC group (n = 6, p = 0.0090; Fig 6I). These findings suggest that CRY2 plays a role in SD-induced cognitive decline in AD mice by inducing CISH expression and function.

## 4. Discussion

The present study demonstrated that sleep deprivation (SD) led to cognitive decline in AD mice and increased the expression of CRY2 in the hippocampus. Inhibition or knockdown of CRY2 in the hippocampus attenuated the development of SD-induced cognitive decline in AD mice, and CRY2 overexpression promoted cognitive dysfunction in AD mice. The CISH/p-STAT1 pathway was involved in the regulation of CRY2 expression during SD. These findings suggest that CRY2 is a promising therapeutic target for SD-induced cognitive decline.

The present study constructed SD models in wild-type (WT) and AD mice. Previous studies showed a significant decrease in ^18^F-FDG absorption in Alzheimer’s patients compared to normal individuals [21, 25]. We used ^18^F-FDG-PET/CT to measure the glucose uptake of neurons and glial cells. This technique is sensitive to synaptic dysfunction, and it is an established imaging technique for the diagnosis of Alzheimer’s disease. SD primarily affected the cerebral cortex and hippocampus of AD mice. Wu showed reduced relative cerebral glucose metabolism in the right amygdala, the right anterior cingulate cortex, and the right posterior cingulate gyrus in insomnia disorder patients compared to healthy controls [26]. The brain ^18^F-FDG metabolism of healthy individuals is higher than insomnia patients in specific areas, including the hypothalamus, hippocampus, amygdala, anterior cingulate cortex, and prefrontal cortex [27]. Our results are not identical to these studies, likely because we studied different species or used different sleep deprivation regimens. Our study showed that sleep deprivation had a significant effect on the left hippocampus of AD mice, but not on the right hippocampus of AD mice. This may be because the left hippocampus is more affected when processing negative emotions [28]. Although WT and AD mice underwent the same duration of SD, only mice in the AD+SD group showed a significant decrease in SUV, which may be because glucose metabolism in AD mice is more sensitive to the effects of sleep deprivation. The behavioral tests showed that sleep deprivation aggravated the cognitive decline of AD mice but had no effect on cognitive function in WT mice. The degree of damage in WT mice was not sufficient to affect cognitive function, but the AD mice showed cognitive decline, which was likely because the brains of these mice contained Aβ as an additional disruptor to synaptic function.

We focused on the mechanisms of SD on synaptic dysfunction in the hippocampus. Previous studies also showed that sleep deprivation caused cognitive decline in AD mice [29, 30], which led to changes in rhythm gene expression [6]. We found that CRY2 was significantly increased in AD mice, which indicated that SD had a greater effect on AD mice. Our results are consistent with Wang, who found significant increases in CRY2 in sleep-deprived mice with another disease [31]. The reason for the different outcomes of the effects of sleep deprivation on WT mice and AD in our study may be that Aβ in the brains of AD mice alters the expression of rhythm genes [32, 33]. We also detected the expression of CRY2 in the dentate gyrus (DG) of the hippocampus, and it was primarily expressed in neurons. Overexpression of CRY2 in the hippocampal DG region of AD mice led to a decrease in cognitive function without SD, and knockdown of CRY2 protected against SD-induced cognitive decline in AD mice.

The JAK/STAT pathway plays an important role in the pathogenesis of AD, and it regulates several CNS functions, including neurogenesis, gliogenesis, synaptic plasticity, and microglial activation [34, 35]. The JAK/STAT pathway is activated during sleep deprivation and leads to atrophy of the masseter muscle [36]. Our study found that SD decreased cytokine-induced SH2-containing protein (CISH) and increased p-STAT1 after SD or overexpression of CRY2. CISH is a suppressor of members of the SOCS family and negatively regulates STAT1 downstream of the growth hormone receptor. Overexpression of CISH in the hippocampal DG region of AD mice improved the SD-induced cognitive decline.

In conclusion, our findings suggest that sleep deprivation upregulated CRY2 in the hippocampus of AD mice, which resulted in synaptic dysfunction by decreasing CISH-mediated STAT1 phosphorylation.

## Supporting information

S1 File (RAR)
